# PREDICTING THE RISK FACTORS OF HYPERTENSION AMONG INDIAN OLDER POPULATION: A MACHINE LEARNING APPROACH

**DOI:** 10.1093/geroni/igad104.1480

**Published:** 2023-12-21

## Abstract

Hypertension, often known as high blood pressure, is a disorder in which the pressure inside the blood arteries remain consistently high. It is a common chronic illness that often occurs along with other morbidities. Risk prediction of hypertension further contributes to the detection, development of interventions, and treatment. The current study used different machine learning models to identify the high-risk factors of developing hypertension using a large-scale survey dataset LASI. A sample of 58,757 collected through the survey aged 45 and above used for model training and testing. We identified 35 risk factors from the literature and then applied feature selection methods to identify only the important ones. Based on the selected features, three models were built i.e., random forest, decision tree, and logistic regression, and the hyper-parameters were optimized on the training set using 10-fold cross-validation. The performance of the models was measured using area under the curve value, precision, recall, and F measure. The random forest classifier outperformed the rest of the two algorithms with an AUC score of 0.64, a precision of 0.65, a recall score of 0.57, and an F1 score of 0.61. Body Mass Index, diabetes, age, type of work, parents having hypertension, and multimorbidity are the primary risk factors for hypertension. The results can be further applied to make algorithms to predict hypertension among an older individuals based on their demographic, health and behavioral factors. Predictive models for hypertension can aid in determining the degree of community interventions required, ensuring a favorable outcome.

